# Albumin-deficient mouse models for studying metabolism of human albumin and pharmacokinetics of albumin-based drugs

**DOI:** 10.1080/19420862.2015.1008345

**Published:** 2015-02-05

**Authors:** Derry C Roopenian, Benjamin E Low, Gregory J Christianson, Gabriele Proetzel, Thomas J Sproule, Michael V Wiles

**Affiliations:** The Jackson Laboratory; Bar Harbor, ME USA

**Keywords:** albumin-conjugates, pharmacokinetics, neonatal Fc receptor, mouse model, human serum albumin, analbuminemia, hypoalbuminemia, transgenic, albumin, TALEN

## Abstract

Serum albumin is the major determinant of blood colloidal osmotic pressure acting as a depot and distributor of compounds including drugs. In humans, serum albumin exhibits an unusually long half-life mainly due to protection from catabolism by neonatal Fc receptor (FcRn)-mediated recycling. These properties make albumin an attractive courier of therapeutically-active compounds. However, pharmaceutical research and development of albumin-based therapeutics has been hampered by the lack of appropriate preclinical animal models. To overcome this, we developed and describe the first mouse with a genetic deficiency in albumin and its incorporation into an existing humanized FcRn mouse model, B6.Cg-*Fcgrt^tm1Dcr^* Tg(FCGRT)32Dcr/DcrJ (Tg32). Albumin-deficient strains (*Alb^-/-^*) were created by TALEN-mediated disruption of the albumin (*Alb*) gene directly in fertilized oocytes derived from Tg32 mice and its non-transgenic background control, C57BL/6J (B6). The resulting *Alb^-/-^* strains are analbuminemic but healthy. Intravenous administration of human albumin to Tg32-*Alb^-/-^ mFcRn^-/-^ hFcRn^Tg/Tg^*) mice results in a remarkably extended human albumin serum half-life of ∼24 days, comparable to that found in humans, and in contrast to half-lives of 2.6–5.8 d observed in B6, B6-*Alb^-/-^* and Tg32 strains. This striking increase can be explained by the absence of competing endogenous mouse albumin and the presence of an active human FcRn. These novel albumin-deficient models provide unique tools for investigating the biology and pathobiology of serum albumin and are a more appropriate rodent surrogates for evaluating human serum albumin pharmacokinetics and albumin-based compounds.

## Abbreviations

HSA: human serum albumin
IgG: immunoglobulin G
FcRn: neonatal Fc receptor
hFcRn: human FcRn
TALEN: transcription activator-like effector nuclease
MSA: mouse serum albumin
ALT: alanine aminotransferase
HDL: high-density lipoprotein
LDH: low-density-lipoprotein
AST: aspartate aminotransferase

## Introduction

Human serum albumin (HSA) is the most abundant protein in blood, typically at 30–50 mg/ml, and has been studied for centuries. Hippocrates first inferred its presence around 400 B.C. observing persistently foamy urine in patients with "dropsy," i.e., edema as the result of kidney disease leading to albuminuria.^1,2^ However, only in recent times have we begun to understand albumin's multiple roles. Due to its abundance in serum, albumin is regarded as the primary controller of total colloid osmotic pressure. In perhaps a less passive capacity, it also serves as a depot and circulatory courier of endogenous compounds, including steroids, fatty acids, thyroid hormones, metabolites (e.g., the antioxidant bilirubin), heme, various minerals and heavy metals, and also of small molecule drugs and their metabolites.^3-5^

The abundance of any circulatory protein is the result of a dynamic balance of synthesis, persistence and elimination. The unusual pharmacokinetic behavior of human HSA and its parallels with IgG were first recognized by Waldman and Strober.^6^ Both immunoglobulin G (IgG) and HSA stand out among serum proteins in their exceptionally long half-lives of ∼21 d in humans. These long half-lives are principally due to active recycling by the neonatal Fc receptor (FcRn). The mechanisms by which FcRn protects and rescues these ligands from intracellular catabolic elimination have been described in detail. ^7-12^

While the medical use of serum albumin has until recently been limited to treating burns, shock and blood loss, awareness of its pharmacokinetics combined with its binding properties has promoted interest in its use as a non-immunogenic vehicle for enhancing the persistence of drugs. Indeed, HSA-drug conjugates, albumin fusion proteins, and non-conjugated forms composed of albumin nanoparticles are already in, or nearing clinical use.^5,13-16^ However, a major limitation to preclinical pharmacokinetic evaluation and development of novel serum albumin-based therapeutics has been the lack of animal models that more faithfully reflect human albumin metabolism. It has been previously observed in mice that serum half-life of administered human albumin was strikingly short (∼2 d; D.C.R, unpublished data, and this publication). This is in contrast to the ∼21 d half-life of HSA observed in humans and hence questions the translational value using mice for modeling HSA pharmacokinetic behavior. The proposed cause for this are is species differences, in which mouse FcRn (mFcRn) binds HSA poorly, and therefore is being outcompeted by the excessive concentrations of mouse serum albumin (MSA) in mice, leading to its short half-life. ^11,17,18^ To begin to address this challenge, we previously created humanized FcRn mouse models in which the native mFcRn heavy chain gene, *Fcgrt* was deleted and subsequently substituted with a human *FCGRT* transgene (hFcRn).^19-21^ These humanized FcRn platform strains have proven utility for pharmacokinetic and preclinical evaluations of chimeric, humanized and human IgG monoclonal antibodies, demonstrating a high correlation to those observed in the cynomolgus monkey and humans.^22,23^ However, as described in our present study, the suitability of these mice for evaluating HSA pharmacokinetics continues to be hampered by competing endogenous MSA, resulting in a HSA half-life only incrementally longer than that found in mice with native FcRn.

Here, we describe the modification and characterization of an improved hFcRn humanized mouse model customized for the investigation of human serum albumin biology and the development of serum albumin-based therapeutics. Using a TALEN targeting approach we disrupted mouse *Alb* directly in fertilized oocytes isolated from the mFcRn^-/-^ hFcRn transgenic mouse strain B6.Cg-*Fcgrt^tm1Dcr^* Tg(FCGRT)32Dcr/DcrJ (abbreviated here to Tg32), and also in its wild type background strain C57BL/6J (B6).^24^ Surprisingly, despite the lack of detectable serum albumin, the resulting *Alb^-/-^* mouse strains are healthy and breed similarly to their parental strains, while exhibiting changes in serum chemistry consistent with analbuminemia. Crucially, upon intravenous (IV) administration of HSA, *Alb^-/-^* hFcRn humanized mice now demonstrate significantly extended pharmacokinetic profiles, with a HSA half-life of ∼24 days, reflecting that found in humans. These novel albumin-deficient mouse models now provide more accurate rodent surrogates not only for evaluating HSA pharmacokinetics and developing albumin-based therapeutics, but also for investigating the biology and pathobiology of serum albumin.

## Results

### Generation of albumin null mouse strains

**Figure 1.**
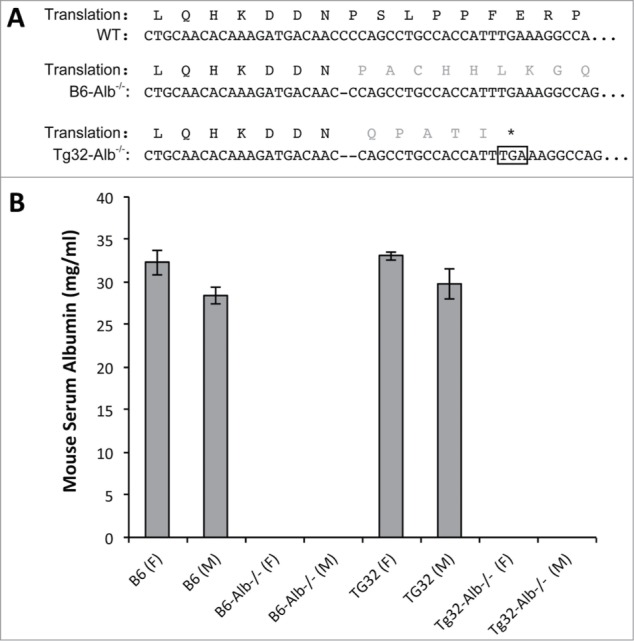
Description of albumin mutations and MSA levels. (**A**) TALEN targeted region of albumin exon 4, DNA and predicted amino acid sequences. Dashes indicate deleted base(s) in the mutant allele and the amino acids in gray illustrate the predicted change from wild type sequence due to the frame shift. In B6-*Alb^-/-^*, TALEN-mediated deletion causes a frame shift leading to a premature stop codon 55 amino acids downstream (not shown). In Tg32-*Alb^-/-^* the mutation leads to a frame shift and a stop codon 5 amino acids downstream (boxed*). (**B**) Mouse albumin concentrations in serum. For each group, 4 males and 4 females were analyzed. MSA concentrations of mice for *Alb*-null homozygotes were below the detection limit of the ELISA (0.2 g/L).

The mouse *Alb* locus consists of 15 exons. To disrupt the gene, exon 4 (Domain 1 ^25^) was targeted using TALEN introduced directly as mRNAs into fertilized oocytes isolated from B6 or Tg32 homozygous matings (see **Fig. 1A**). The resulting offspring were screened to identify founders with deletions. These founders were backcrossed to B6 or Tg32 as required to maintain a fixed background and intercrossed to obtain lines homozygous for specific mutation events. A B6 line carrying a 1 base pair (bp) deletion (*em8*, GRCm38.p2 C57BL/6J base 90464113) and a Tg32 line with a 2 bp deletion (*em12*, GRCm38.p2 C57BL/6J bases 90464113 and 90464114), both in exon 4, were chosen for further study. In both cases, exon 4 deletions were predicted to disrupt albumin translation due to frame shifts resulting in premature stop codons (for the B6 *Alb* mutation, 55 amino acids downstream, and for the Tg32 *Alb* mutation, 5 amino acids downstream of the deletion (**Fig. 1A**). Both strains were homozygosed for the disruptive mutation and designated C57BL/6J-*Alb^em8Mvw^*/MvwJ, abbreviated here to B6-*Alb^-/-^*^,^ and for the Tg32 *Alb* mutation, B6.Cg-*Alb^em12Mvw^Fcgrt^tm1Dcr^* Tg(FCGRT)32Dcr/MvwJ, abbreviated here to Tg32-*Alb^-/-^*.

To determine if B6-*Alb^-/-^* and Tg32-*Alb^-/-^* strains are albuminemic, MSA concentrations were measured by ELISA. Sera samples from heterozygous animals from both strains showed a ∼30% reduction in MSA levels compared with the wild type counterparts (data not shown), whereas MSA in homozygosed B6-*Alb^-/-^* and Tg32-*Alb^-/-^* strains was not detectable above background levels (≤0.2mg/L) of the ELISA (**Fig. 1B** and **Table 1**) as expected due to the nature of the mutations.

**Table 1.** Blood chemistry for *Alb* null strains B6-*Alb^−/−^* and Tg32-*Alb^−/−^*, control background mice, and comparative data for humans

		♂Background B6 (Standard Deviation)		♂Background Tg32 (Standard Deviation)		Human Comparison		
Serum Component	Unit	Parental	Albumin Null	Parental	Albumin Null	Normal range	Analbumin-emic	Reference
Albumin	g/L	28.4 (0.99)	≤0.2	29.8 (1.84)	≤0.2	30–50g/L	0 to ≤10	^1,^^26^^,^^28,^^33^
Alanine aminotransferase (ALT)	IU/L	31.2 (4.57)	65.0 (19.4)	30.9 (2.23)	43.0 (9.49)	7–56 U/L	unchanged	^27^
Aspartate aminotransferase (AST)	IU/L	42.8 (5.06)	103.8 (28.1)	48.0 (4.69)	83.8 (34.51)	3–35 U/L	unchanged	^27^
Bilirubin (total)	mg/dL	0.188 (0.010)	0.085 (0.010)	0.170 (0.010)	0.058 (0.001)	0.4–1.9 mg/dL	n/d	^37^
Cholesterol (total)	mg/dL	121.5 (12.5)	164.8 (7.5)	126.75 (2.75)	147.25 (44.91)	<5.17 mmol/L	6 mmol/L	^27-29^^,^^32, 33^
Calcium	mg/dL	10.25 (0.25)	8.93 (0.05)	10.45 (0.13)	8.75 (0.13)	2.15–2.55 mmol/L	1.42 mmol/L	^26^, ^28^^,^^30^, ^33^
High-Density-Lipoprotein cholesterol (HDL)	mg/dL	103.18 (9.45)	131.3 (4.71)	104.3 (2.94)	105.1 (51.65)	1.03–1.55 mmol/L	1.88 mmol/L or unchanged	^26^^,^^29-33^
Iron	μg/dL	174.75 (21.11)	204.25 (12.89)	142.75 (10.90)	224.00 (21.86)	70–160 μg/dl	55 μg/ml	^28^, ^41^
Lipase	U/L	89.25 (2.36)	127.25 (19.43)	85.75 (11.84)	117 (17.66)	12–70 U/L	n/d	^42^
Low-density-lipoprotein (LDH)	mg/dL	4.025 (0.70)	8.325 (1.53)	5.35 (0.94)	20.075 (8.79)	<2.59 mmol/L	2.66 mmol/L	^26^^,^^28-30^^,^^32^
Nonesterified fatty acids (NEFA)	mEg/L	1.625 (0.31)	1.050 (0.08)	1.685 (0.141)	0.96 (0.400)	0.2–0.7 mEq/L	Same or unchanged	^1^, ^26^^,^^31^
Total protein	g/dL	5.625 (0.19)	3.825 (0.17)	5.8 (0.245)	3.725 (0.340)	6–8g/dl	5.6g/dl	^26-30^
Triglycerides (total)	mg/dL	127 (12.38)	213.75 (58.22)	101.75 (10.31)	131.75 (57.80)	<1.69 mmol/L	4.47mmol/L or unchanged	^28-31^

*Serum blood chemistry using 4 males at 7–8 wks of age comparing with published data for normal and analbuminemic humans. Significant differences in mouse serum values heighted *p* value <0.05 shaded light gray, *p* value <0.0001 dark gray.

### Blood chemistry and gross histopathology

We performed blood chemistry comparisons of both *Alb^-/-^* strains and their parental *Alb^+/+^* counterparts to identify alterations caused in the absence of albumin. Using mice at 7–8 wks of age data obtained are, in general, consistent with an analbuminemic state (**Table 1**). Accompanying the lack of detectable serum albumin, we found, small but significant reductions in total serum protein, suggesting only partial compensation by other major serum proteins.^1,26-30^ Also noted was a decrease in nonesterified fatty acids. In contrast, *Alb* null mice showed elevated levels of total bilirubin, lipase, calcium and alanine aminotransferase (ALT), and variable increases in total cholesterol, high-density lipoprotein (HDL), low-density-lipoprotein (LDH), aspartate aminotransferase (AST), iron and total triglycerides. These data are consistent with generalized hyperlipidemia and other changes reported for human analbuminemia. ^1,26,28-33^

Despite these serum changes, both *Alb^-/-^* strains are healthy, maintaining normal body weights and breeding similarly to their *Alb^+/+^* parental strains. Gross examinations by necropsy of ∼8 wks mice from both *Alb^-/-^* strains were unremarkable, lacking overt differences compared with the background strains, B6 and Tg32. This included no evidence for edema, which is sometimes reported in humans with analbuminemia.^29^ Liver weight to body weight ratios were also not significantly different between control and *Alb^-/-^* strains. Histological analysis of livers showed no detectable lesions. Oil Red O staining for lipid accumulations in the heart and aorta, as indicators for coronary disease showed no irregularities (data not shown).

### HSA recycling

**Figure 2.**
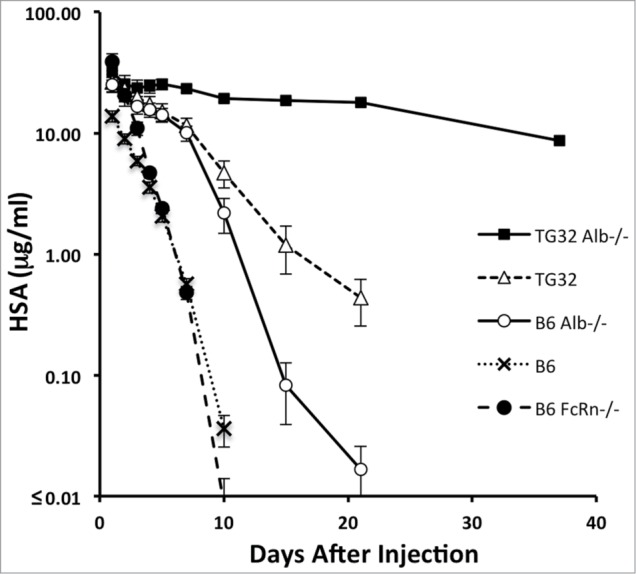
Species differences in FcRn and albumin deficiency influence the persistence of HSA. Seven mice of each strain were pre-bled 14 d before administering HSA or HSA and hIgG to establish baselines. Five mice of each group were then injected IV with HSA (10 mg/kg) and 2 were injected with 10 mg/kg HSA + 5 mg/kg hIgG. All mice were then bled post injection at 10 min, 12 h, 24 h, 48 h, 72 h, 96 h, 120 h and then at 7 d, 10 d, 15 d, 21 d and 37 d. Mean HSA concentrations in mg +/- standard error of the mean (SEM) (*n* = 7). In some cases, the SEM is smaller than the symbols.

Human serum albumin clearance studies were performed to determine the effect of endogenous MSA on the pharmacokinetic behavior of administered human albumin. Purified HSA was injected intravenously (IV) at 10 mg/kg into 7 age and sex-matched Tg32-*Alb^-/-^*, B6-*Alb^-/-^*, B6.129×1-*Fcgrt*^tm1Dcr^/DcrJ (abbreviated here to B6-*mFcRna^-/-^*), Tg32 and B6 mice. Two mice of each cohort also received 5 mg/kg human IgG in the same IV injections. Serial serum samples were collected and HSA concentrations were determined; data are summarized in **Figure 2** and **Table 2**.

**Table 2.** Estimated beta-phase half-lives of IV introduced HSA into parental and derived *Alb*-null mouse strains

Strain	Description and genotype	t_1/2_ HSA [days] (n = 7)	SEM [days]
B6	C57BL/6J	2.6	0.1
B6-mFcRn^−/−^	B6 murine FcRn null	2.1	0.1
B6-Alb^−/-^	B6 murine Alb null	4.2	0.6
Tg32	B6 murine FcRn null, human FcRn transgenic ^+/+^	5.8	0.5
Tg32-Alb^−/-^	B6 murine FcRn null, murine Alb null, human FcRn transgenic ^+/+^	24.1	2.8

As expected, B6-*mFcRn^-/-^* mice cleared HSA rapidly (half-life 2.1 days), while the half-life was minimally extended in B6 mice (half-life 2.6 days). In B6-*Alb^-/-^* mice, HSA half-life was significantly increased (half-life 4.2 days) indicating that in the absence of competing endogenous MSA mFcRn can recycle HSA more efficiently. A further extension was observed in Tg32 mice with MSA (half-live of 5.8 days), suggesting that hFcRn protects HSA more efficiently under conditions in which endogenous MSA is competing compared to mFcRn. Finally and most strikingly, Tg32 *Alb^-/-^* mice demonstrate an HSA half-life of 24.1 days, retaining ∼27% of the initial maximum concentrations of HSA at 37 d The monophasic log-linear pharmacokinetic profiles observed were consistent with a lack of immunogenicity and anti-HSA antibodies; such effects would result in 2-phase clearance kinetics resulting in a precipitous loss of HSA typically on days 5–7 once anti-HSA antibodies develop. Overall, the data clearly show that hFcRn protects HSA quite efficiently, especially under conditions in which endogenous MSA is absent.

**Figure 3.**
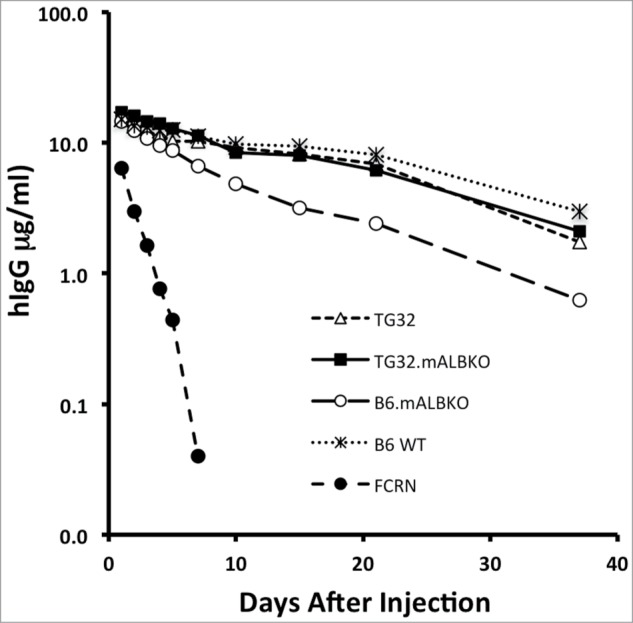
Species differences in FcRn and albumin deficiency influence the persistence of hIgG. Two mice of each of the 7 strains were pre-bled 14 d before administering HSA and hIgG to establish baselines. Mice were then injected IV with 10 mg/kg HSA + 5 mg/kg hIgG. All were then bled post injection at 10 min, 12 h, 24 h, 48 h, 72 h, 96 h, 120 h and then at 7 d, 10 d, 15 d, 21 d and 37 d. Mean percent of starting level of hIgG concentration per group (*n* = 2) is shown, variation between replicates was not significant.

To confirm that IgG recycling was not altered in *Alb^-/-^* mice, serum hIgG levels were determined for the 2 mice of each strain that had received hIgG + HSA (**Fig. 3**). Human IgG levels did not differ in *Alb^-/-^* and FcRn expressing control strains, while B6-*mFcRn^-/-^* mice showed the expected short half-lives for both IgG and HSA due to the complete lack of FcRn recycling. These data are in agreement with those previously published showing that IgG and serum albumin are recycled by FcRn non-competitively. ^8,11^

## Discussion

The goal of this study was to develop and characterize mouse models designed to facilitate the study of albumin biology and for use in the development of albumin-based compounds. Using TALEN gene editing techniques, we directly disrupted the *Alb* gene in zygotes derived from B6 and Tg32 mouse strains. This direct modification approach exemplifies the power of targeted nuclease gene editing techniques to sequentially manipulate and refine the genome of "platform" mouse models. By direct genetic modification, we bypassed time-consuming embryonic stem cell methods, or time-consuming breeding to develop mouse models customized for the preclinical development and assessment of albumin-based therapeutics.

Previously, facets of serum albumin biology and pathobiology have been uncovered indirectly based on hypoalbuminemic states caused by hepatic insufficiencies, nephrotic syndrome, and protein-losing enteropathies and through limited studies of familial human analbuminemias. Although a spontaneous analbuminemic Nagase rat model also exists, this model has not been readily available.^34,35^ Furthermore, this rat model is limited for studies in the context of rodent specificity of FcRn and has not been further genetically manipulated. Also to date, no spontaneous analbuminemic or genetically engineered *Alb* knockout mouse has been described. However, our studies presented here do parallel observations in analbuminemic humans and rats, with where the absence of this dominant serum protein in mice is surprisingly innocuous.^36^ We found that *Alb^-/-^* mice have only slightly lower total serum proteins, suggesting partial compensation by other primarily liver-derived serum proteins. Other serum parameter changes observed in *Alb^-/-^* mice are consistent with secondary effects that include a generalized hyperlipidemic state, but with an absence of any overt histological or clinical lesions as detected at 7–8 weeks of age. Not previously observed in humans but reported in Nagase rats, bilirubin levels are strikingly reduced in both *Alb^-/-^* strains developed here (p>0.0001). This is consistent with data suggesting that albumin is necessary for the production of protein-bound bilirubin (Bδ).^37^ Longer term studies are needed to determine if *Alb^-/-^* mice develop more significant phenotypes at a later age or when exposed to drugs whose toxicity is normally buffered by serum albumin.^38^

A major outcome of our FcRn studies here are new insights into the species differences in the pharmacokinetic behavior of serum albumins. In a phenomenon referred to as the concentration–catabolism effect,^6^ the efficiency by which FcRn protects its ligands from catabolic elimination is governed by its receptor occupancy. HSA binds mFcRn quite weakly compared with MSA, with reported KDs of 90.0 mM and 9.3 mM, respectively at acidic pH, and is therefore more likely to be outcompeted by the excessive concentrations of MSA in mice.^18^ This dynamic, involving both affinity and serum concentration differences, may account for the marginal half-live extension of HSA (from 2.1 to 2.6 days) comparing *mFcRn^-/-^* with *mFcRn^+/+^* mice and its increase to 4.2 d in *mFcRn^+/+^* analbuminemic mice (**Table 2**). The HSA half-life was further increased to 5.8 d in FcRn-humanized *Alb^+/+^* mice and achieved a striking HSA half-life of 24 d in FcRn-humanized *Alb^-/-^* mice (**Table 2**). While human FcRn has a stronger binding affinity for both ligands, with reported KDs of 0.8 mM for MSA and 4.5 mM for HSA,^18^ the half-life increases caused by FcRn-humanization suggest that HSA is more efficiently protected by hFcRn. Overall the results show that species differences both in albumin and FcRn can substantially confound the pharmacokinetic analysis of HSA and that *Alb^-/-^* hFcRn transgenic mice may more reliably model the pharmacokinetic behavior of human albumin-based therapeutics.

A second major outcome is the development and characterization of novel analbuminemic mouse models. Further study of these models promises to extend the understanding of the biology and pathobiology of serum albumin and human hypoalbuminemic states and facilitate the development of human serum albumin-based therapeutics.

## Materials and Methods

### Targeted gene disruptions

A pair of TALENs were designed using ZiFit Targeter software and were assembled using the REAL FAST methodology.^39,40^ The TALEN pair targeted 5'-TTTCCTGCAACACAAAGA tgacaaccccagcctg CCACCATTTGAAAGGCCA-3′ (TALEN recognition sequences capitalized, intervening sequence in lowercase). Capped and tailed mRNA encoding each TALEN was prepared using the AmpliCap-Max™ T7 High Yield Message Maker Kit (Cellscript, Cat. No. C-ACM04037) and the A-Plus™ Poly(A) Polymerase Tailing Kit (Cellscript, Cat. No. C-PAP5104H). TALEN mRNA, each at 10 ng/μl, was injected into the cytoplasm of B6 or Tg32 zygotes, transferred into pseudopregnant females and brought to term. Offspring were screened for deletions by PCR using primers flanking the targeted region (followed by sequencing from both sides (5'CCTCAAGGCCATTAGCTCTTTGCT and 5'TACACGCACATGCACACAAATGCC resulting in a 883bp product with wild type derived DNA). Founder animals carrying deletions were bred to the parental strain (B6 or Tg32) to maintain a fixed genetic background. The resulting offspring were reanalyzed for the mutations and intercrossed to produce lines homozygous for the *Alb* mutations. One mutant line derived from each of the backgrounds (B6 and Tg32) was selected for further study. These strains are available from The Jackson Laboratory.

### Experimental animals

Mice were provided with acidified water, a 5K54 diet (LabDiet, St. Louis, MO) and housed in cages exposed to a 12-hour light-dark cycle in The Jackson Laboratory Research Animal Facility. All mice were treated in accordance with the Animal Care and Use Committee at The Jackson Laboratory. Mice developed and used in this study are described in **Table 3**.

**Table 3.** Mouse strains used and created in this study

			Genotype		
Short Name	Full Strain Name	JAX Stock Ref #	m*Alb*	m*Fcgrt*	h*FCGRT*
B6	C57BL/6J	000664	wt	wt	null
B6-Alb^−/−^	C57BL/6J-*Alb^em8Mvw^*/MvwJ	025200	-/-	wt	null
B6-mFcRn^−/−^	B6.129×1-*Fcgrt^tm1Dcr^*/DcrJ	003982	wt	-/-	null
Tg32	B6.Cg-*Fcgrt^tm1Dcr^* Tg(FCGRT)32Dcr/DcrJ	014565	wt	-/-	Tg/Tg
Tg32-Alb^−/−^	B6.Cg-*Alb^em12Mvw^Fcgrt^tm1Dcr^* Tg(FCGRT)32Dcr/MvwJ	025201	-/-	-/-	Tg/Tg

All mice are available from The Jackson Laboratory.

### Genotyping assays for albumin mutant mice

*Alb* knockout mice used in this study were genotyped using PCR, the product was then sequenced to identify the 1-base (B6-*Alb^-/-^*) or 2-base (Tg32-*Alb^-/-^*) deletions. Primers used for both PCR and sequencing produce an 883bp amplicon from the unaltered allele; 5′-CCTCAAGGCCATTAGCTCTTTGCT and 5′-TACACGCACATGCACACAAATGCC.

### Serum and plasma collection

For ELISAs, 25 μl whole blood was collected from the retro-orbital sinus using non-heparinized capillary tubes (Drummond, cat. 1–000–0250), mixed with heparin (1 μl at 10 U/μl) and maintained on ice before centrifugation (10,000 x g for 5 min at 4°C). The top 10 μl of plasma was added to storage buffer (90 μl, 50% glycerol in phosphate buffered saline (PBS)), mixed and kept at −20°C before use. For blood chemistry, ∼200 μl whole blood was obtained via submandibular cheek puncture using non-heparinized capillary tubes and stored in Microtainer tubes (Becton Dickinson, cat. 365956), allowed to coagulate at room temperature between 30 min and 3 hours, centrifuged (20,000 x g for 10 min) and supernatant (serum) collected for analysis.

### Blood chemistry

Serum was collected from non-fasting male mice (*n* = 4), age 7 wks (+/- 3 d) from each of the *Alb* knockout strains as well as respective strain backgrounds (B6, B6-*Alb^-/-^*, Tg32, Tg32-*Alb^-/-^*Blood chemistry was performed using a Beckman AU680 analyzer to screen for the following parameters: ALT, AST, cholesterol, triglycerides, lipase, LDLD, HDLX, NEFA, calcium, total protein, total bilirubin, CK, and iron.

Data obtained was subject to a one-way analysis of variance (ANOVA) using "strain" as the only factor was performed on the B6 vs. B6-*Alb^-/-^*and TG32 vs. TG32-*Alb^-/-^* data sets. No data transformations we applied prior to the one-way ANOVA. Phenotypes with Strain p-values <0.004 (Bonferroni adjusted p-value) were deemed to be statistically significant. The Bonferroni adjusted p-value was used to account for the multiple phenotypes tested.

### Albumin and IgG clearance

Seven male mice aged 10–13 wks from each strain (B6, B6-*Alb^-/-^*, B6-*mFcRn^-/-^*, Tg32, Tg32-*Alb^-/-^*) received a single dose via tail-vein injection of either HSA (Plasbumin, Talecris, 13533–684–16) alone, or HSA admixed with human IgG (Baxter, cat. Gammagard S/D). Five animals received the injection of albumin alone, at a concentration of ∼10 mg/kg, while 2 were injected with an HSA (∼10 mg/kg) + hIgG (∼5 mg/kg) admixture. Two weeks prior to IV injection, serum was collected. Post IV injection, mice were bled at 10 minutes, 12 h, and then 24, 48, 72, 96, and 120 h, then at 7, 10, 15, 21 and 37 d. Serum concentrations were determined by ELISA. Beta-phase HSA half-lives were calculated for each individual mouse and then averaged across the strain.^19^

### Quantification of HSA, MSA and hIgG

HSA was detected using an antigen-based ELISA. Briefly, 96-well plates (Greiner Bio-One, cat. 655061) were coated with 5 μg/ml rabbit anti-HSA antibody (US Biological, cat. A1327–46), blocked with 5% bovine serum albumin (Sigma-Aldrich, cat. A7284) in PBS and incubated with appropriately diluted plasma samples (1:200). Detection used alkaline phosphatase conjugated goat anti-HSA antibody previously cross-adsorbed against bovine, mouse, and pig albumins (Bethyl Laboratories Inc.., cat. A80–229AP). Activity was reported at 405 nm after development with colorimetric *p*-nitrophenyl phosphate substrate (Amresco Inc.., cat. 0405), used at 1 mg/ml. For quantification, a standard curve was prepared using serial dilutions prepared from HSA (Talecris, cat. 13533–684–16). MSA was detected using a commercial ELISA kit (Genway Biotech Inc.., cat. GWB-282C17). Human IgG was detected using ELISA, prepared as described above for HSA, with the exception that capture and detection antibodies were Mouse Anti-Human IgG Fc-UNLB (Southern Biotech, cat. 9040–01) and Mouse Anti-Human Kappa-AP (Southern Biotech, cat. 9230–04). The IgG standard curve was prepared using serial dilutions prepared from Human IgG (Baxter, cat. Gammagard S/D 1501375).
